# Benefits Derived from Washing in Cold Water

**Published:** 1881-10

**Authors:** 


### BENEFITS DERIVED FROM WASHING IN COLD WATER.

As in most things, so in washing, there are two ways of doing it. Some people take a bath who have but a dim idea of washing themselves, and are vexed and annoyed when told the result is not happy.

It is a well-known fact, but rarely remembered, that the skin is one of the great safety-valves of the human machine—that the millions of little perspiratory tubes with which it is pierced throw out from the inner body an average amount of thirty-three ounces of greasy refuse and worn-out material in an hour in the shape of invisible perspiration and in the same time often as much as two or three pounds in visible perspiration. Should these tubes or pores be allowed to remain choked with their own secretions, the refuse matter is thrown back into the other great corporeal scavengers—the lungs, stomach, liver or kidneys. Thus, it stands to reason that a careful and general cleansing of the skin is absolutely necessary to the life and well-being of the iudividual at least once in twenty-four hours, and few people who rejoice in the comfort of cleanliness will feel that it is secured under this amount of washing. And we would also here point out the mere passage of water, especially cold water (*e. g.*, what is ordinarily called a sponge bath), does not cleanse. In fact, it rather has a tendency to close the pores which, like delicate flowers, shut up to a cold current of wind or water. We therefore recommend, as warm or tepid water tends to open the pores, to use that with the course of soap scrubbing (not an unreasonable friction) which should precede the universal sponging. This last may be done with cold water, which certainly invigorates and braces the system when followed by a reactionary glow of warmth. Should this not occur it is unwise to use it, and warm must be substituted, especially in the cases of children, who by ignorant mothers are often forced into cold water (from which they have not a sufficiently active circulation to recover) as part of that much-abused system of "hardening," which nine times out of ten ends in "hardening" the child off the face of the earth, or checking its growth.

"Hardening," it must be understood, should be strengthening, not "roughing," and many people with the best intentions think, very erroneously, that to make a child strong consists in causing it to undergo more physical hardships than they, with their perfectly-matured strength and age, would dream of doing.

As people in conclusions generally rush to extremes, it might be well here to remark that we do not at all recommend coddling; but no wise mother will put her young children into quite cold water in winter time, nor with a cold, and, above all, will never allow them to be washed and bathed in a draught, on the same principle of consistency that plenty of fresh air is good, when it is not damp or foggy, but draughts are most injurious.

